# Still little evidence sex differences in spatial navigation are evolutionary adaptations

**DOI:** 10.1098/rsos.231532

**Published:** 2024-01-17

**Authors:** Connor M. Hults, Richard C. Francis, Edward K. Clint, Winter Smith, Elliott R. Sober, Theodore Garland, Justin S. Rhodes

**Affiliations:** ^1^ Department of Psychology, University of Illinois, 603 E. Daniel St., Champaign, IL 61820, USA; ^2^ Richardcfrancis.com; ^3^ Department of Humanities and Social Sciences, Oregon Institute of Technology, Klamath Falls, OR, USA; ^4^ Department of Philosophy, University of Wisconsin, Madison, WI, USA; ^5^ Department of Evolution, Ecology, and Organismal Biology, University of California, Riverside, CA, USA

**Keywords:** adaptation, home range, spandrel, sexual dimorphism, spatial cognition

## Abstract

A putative male advantage in wayfinding ability is the most widely documented sex difference in human cognition and has also been observed in other animals. The common interpretation, the sex-specific adaptation hypothesis, posits that this male advantage evolved as an adaptive response to sex differences in home range size. A previous study a decade ago tested this hypothesis by comparing sex differences in home range size and spatial ability among 11 species and found no relationship. However, the study was limited by the small sample size, the lack of species with a larger female home range and the lack of non-Western human data. The present study represents an update that addresses all of these limitations, including data from 10 more species and from human subsistence cultures. Consistent with the previous result, we found little evidence that sex differences in spatial navigation and home range size are related. We conclude that sex differences in spatial ability are more likely due to experiential factors and/or unselected biological side effects, rather than functional outcomes of natural selection.

## 1. Introduction

Sex differences in humans and other animals are ubiquitous, morphological, physiological and behavioural. A common practice is to consider any sex difference a product of natural selection that serves a specific function for one or the other sex. However, sex differences can arise for non-adaptive reasons, such as by-products of sex physiology or through phenotypic plasticity [1]. The tendency to explain sex differences as products of natural selection is especially common in evolutionary psychology, where there is a long-standing preoccupation with cognitive sex differences [2–15].

Much of this research has focused on spatial cognition, measured in diverse ways. After hundreds of studies and more than a few meta-analyses [8,16–20], we are left with this picture: males outscore females in a statistically significant way in many spatial tasks, to varying degrees, with a small to moderate meta-analytic effect size in tasks directly related to navigation (0.34–0.38; [19]) as defined by convention in interpreting Cohen's *d* [21]. Adaptive explanations generally relate the male advantage in spatial tasks to sex differences in home range size, hereafter referred to as the sex-specific adaptation hypothesis [22]. In animals in which males have larger home ranges than females, it is commonly hypothesized that males experience more selection for wayfinding skill than females. Three adaptive explanations for the difference in home range size have received much attention in the literature, each hypothesized to drive sex differences in navigation, one related to sexual selection, two not.

On the sexual selection account, in polygynous—but not monogamous species—males must range farther than females to maximize reproduction [2,11–13,15]. The second adaptive explanation attributes the sex differences in home range size to differences in foraging behaviour [4–6]. The third is that selection primarily acts on females. In this version, reduced spatial ability is hypothesized to reduce female mobility, which conserves energy and avoids danger during critical reproductive periods [13,14]. This proposal may conflate cognition and motivation. All three hypotheses have been applied to humans. Note that all three hypotheses assume that substantial sex differences for ancestral human home range size existed, though supporting evidence from contemporary subsistence populations is equivocal [23–25].

By far, the most reliable sex differences in spatial cognition come from timed tests of three-dimensional mental rotations in humans (in untimed tests, the sex differences are diminished; see [26]). Though some find that mental rotation correlates with real-world differences in wayfinding ability among individuals [5,7,27–29], the magnitude of the sex difference is much greater in the former, contrived task (e.g. Cohen's *d* = 0.56 [20] or 0.67 [17]). Given the smaller effect sizes for actual wayfinding mentioned previously, it is arguable whether there is a phenomenon here worth addressing in an evolutionary context, adaptationist or otherwise.

Moreover, from an evolutionary perspective, there are *a priori* grounds to doubt whether sufficient conditions were met for natural selection to produce a sexual dimorphism in spatial cognition driven by differences in ranging behaviour. In general, selection on a complex trait in one sex will cause a correlated response in the other unless what is good for the gander is bad for the goose, i.e. there is antagonistic selection [30–32]. This is because typically there are many genes that influence a complex trait such as spatial ability, and these are not restricted to the sex chromosomes or activated only by sex-specific physiology (although sex-specific physiology may affect their expression to some degree). Thus, if selection in males favours certain alleles, then their female offspring will also inherit them. Although it is possible they would not express the same way, that would be because they are in a different genetic background, rather than because of selection *per se*. Thus, it is not sufficient to demonstrate a greater potential fitness advantage for skilful wayfinding in males than in females. It must also be demonstrated that there is a fitness disadvantage for females; otherwise, females would inherit the trait through their (mostly) shared genomes [30–32]. Although some argue that there is a cost to females for superior wayfinding with respect to energy conservation and survival [13,14], a test of this hypothesis failed to support it, albeit with a small sample size [33]. Assuming wayfinding ability has no fitness cost for females, selection for enhanced wayfinding ability in males should cause a correlated response in females [34], although possibly not to the same degree as the direct response in males.

In the absence of sexually antagonistic selection, the most likely explanation for sex differences in cognitive performance in humans in Western cultures has always been that, from an early age, males and females are socialized in sex-specific ways that entail differential engagement in activities related to spatial cognition. We will hereafter refer to this alternative hypothesis as phenotypic plasticity, consistent with the general usage of this term (e.g. [35]). Thus, men may perform slightly better on average than women on spatial navigation-related tasks because, on average, they are more experienced in similar tasks. This is consistent with the undeniable fact that performance is trainable, and experience leads to better performance.

Recent data exploring sex differences in home range size and spatial navigation in human subsistence cultures provide strong support for the plasticity hypothesis [24,25]. Populations in which males engage in foraging to a greater extent, such as the Temne [36], settled Hadza [37], Twe and Himba [33,38], show a male advantage in spatial orientation. By contrast, populations where both sexes travel long distances to forage, such as the Eskimo [36], mobile Hadza [37], Tsimane [25,39] and Mbendjele BaYaka [24], show no difference between the sexes. Although these patterns might result from natural selection over tens of thousands of years, socio-cultural factors related to spatial experience probably make a substantial contribution. The Hadza are a particularly instructive example [37]. Within the same population, a male advantage in pointing tasks was found in the Mangola camps, in which males are more mobile than females, but not in the bush camps, in which both males and females are highly mobile. Note, however, that this was a small study and the location-by-sex interaction for the pointing task was not statistically significant.

Cultural differences, irrespective of sex, also support the phenotypic plasticity hypothesis, even when the cultures being compared are WEIRD (Western, educated, industrialized, rich, democratic). In a comparison of wayfinding between residents of Salt Lake City, Utah and Padua, Italy, the Paduans fared better in wayfinding skills than the Utahns [40]. One salient difference between the two environments is that, like most American cities, Salt Lake City is a grid whereas, in common with most old European cities, Padua is not.

Despite the likelihood that difference in navigation in humans is mainly a function of plasticity, the allure of the sex-specific adaptation hypothesis has inspired many non-human animal studies. The advantage of the animal studies of sex differences in navigation is that usually the animals are kept in standard cages, thus restricting the environmental space to be the same size for both males and females, and hence removing the confound of phenotypic plasticity. Moreover, multiple species displaying different degrees of sex differences in home range size can be included. In this case, a positive correlation between sex differences in home range size and spatial navigation would constitute evidence for adaptation. Unfortunately, most of the animal studies that have been conducted included only two species (e.g. [12,15,41–43]). In testing for adaptations, two-species comparisons have long been shunned in mainstream evolutionary biology [44]. Any two species will differ in countless traits, and by chance alone, the focal trait will differ in the predicted direction 50% of the time. Moreover, more than two data points are needed to establish a correlation.

In 2012, we published a comparative study of 11 species (including humans) and found no significant correlation between sex differences in home range size and spatial navigation, i.e. no support for the sex-specific adaptation hypothesis [1]. However, in 8 of the 11 species, males tended to perform better than females in spatial tasks in a pattern unrelated to home range size. We concluded the sex difference could be a side effect of sex-specific aspects of physiology rather than an evolutionary adaptation (see Discussion for elaboration on the relationship between physiology and spatial cognition). Hereafter, we refer to this hypothesis as the spandrel hypothesis after Gould & Lewontin [45]. In this view, selection on one trait will often result in by-products (i.e. spandrels) of no functional significance, or at least none under direct selection. Sex steroids, for example, serve obviously adaptive roles in sexual differentiation, not least the genitalia in mammals. But they can have unselected side effects, such as acne and male patterned baldness [1,46,47]. Unlike the sex-specific adaptation hypothesis, the spandrel hypothesis predicts no correlation between home range size and spatial navigation, but, very often, significantly different performance between the sexes one way or the other.

The present paper serves to expand on the 2012 study in four key ways: (i) increasing statistical power by including data from 10 new species, including a species in which females have a larger home range; (ii) better addressing the unique ecological factors within each species; (iii) analysing and reviewing data from human subsistence cultures, much of which has been published since the 2012 paper; and (iv) addressing criticisms of the earlier work.

## 2. Methods

### 2.1. Selection of studies

We compiled home range size and spatial navigation data from 66 studies and 21 species (electronic supplementary material, table S1), nearly double the numbers in Clint *et al*. [1]. Species included the Asian small-clawed otter (*Aonyx cinereus*) [15], brilliant-thighed poison frog (*Allobates femoralis*) [48], California mouse (*Peromyscus californicus*) [43,49], chimpanzee (*Pan troglodytes*) [50–53], cuttlefish (*Sepia officinalis*) [54], deer mouse (*Peromyscus maniculatus*) [43,55–57], diablito poison frog (*Oophaga sylvatica*) [48], dyeing poison frog (*Dendrobates tinctorius*) [48], European rabbit (*Oryctolagus cuniculus*) [58–60], giant panda (*Ailuropoda melanoleuca*) [15,61], horse (*Equus caballus*) [62–64], human (*Homo sapiens*) [5,23–25,33,65–78], meadow vole (*Microtus pennsylvanicus*) [12,79–81], mouse (*Mus musculus*) [82–87], Natal mole-rat (*Cryptomys hottentotus*) [88], pine vole (*Microtus pinetorum*) [12], prairie vole (*Microtus ochrogaster*) [42,89–91], rat (*Rattus norvegicus*) [92–96], rhesus monkey (*Macaca mulatta*) [97–99], rusty crayfish (*Orconectes rusticus*) [100,101] and Talas tuco-tuco (*Ctenomys talarum*) [102–104].

Selection criteria were mostly consistent with Clint *et al*. [1]: adult animals, original data, provided data separately for males and females, used uncontroversial measures that were either common in the literature or could reasonably be expected to relate to navigational ability based on the ecology of the species, and had raw data or means available. If the authors did not specify that animals were adults but included ages or weights, standards in the literature were used to determine their stage in development. Data that could not be limited to adults were excluded. When raw data or means were unavailable, but the sex difference was non-significant, the sex difference index (see next section) was set to zero. Note that we also included analyses that excluded such studies where effect sizes were unavailable (see below and electronic supplementary material, table S2).

One significant divergence from the methods used in the original analysis relates to the time of year when home range data were collected. Clint *et al*. [1] confined their home range studies to those in which year-round data were available to allow comparison among species with and without a pronounced breeding season. In contrast, to better account for the unique ecological considerations and predictions for each species, we limited home range data to studies confined to the period in which the greatest sex difference was expected (i.e. breeding season) or included year-round data, assuming that the period of greatest sex difference would be captured. Data obtained outside of the breeding season, when the sex difference in home range or cognition was expected within the breeding season, were excluded. For spatial ability studies, we excluded data obtained outside the period in which the sex differences were expected. Note that these considerations applied to the three frog species, deer mouse, giant panda and meadow vole. All the other species had no pronounced breeding season or no known changes in spatial behaviour within their breeding season.

In the Talas tuco-tuco, home range data on two populations with different ecologies exist. Necochea and Mar de Cobo populations differ in body size, the presence of dominance hierarchies and degree of polygyny [105]. Sex differences in home range, mating season or not, differ between the populations. However, it is unclear why, and no cognition data exist to determine the state of sex differences in spatial navigation between these two populations. Therefore, we limited the home range data to the Mar de Cobo population, as this was the only one for which we had cognitive data. Note that it was not always possible to include home range and spatial navigation data from the same population within a species (e.g. the chimpanzee).

For non-humans, most home range measurements were collected through field observation, radio-tracking or trapping. Additional measurements included powder-tracking for the mouse species, harmonic direction-finding in three species of frogs, locomotor activity as a proxy for the Asian small-clawed otter and cuttlefish, and various survey and mapping methods for humans. Clint *et al*. [1] noted that data from the rhesus monkey and horse are unavailable as they are generally studied in groups, which is still the case at present. However, they argued that ‘it is broadly agreed that both are highly social animals and that males and females remain in close proximity to each other for the vast majority of their lives' [62,63,99], so a sex difference index of zero was used.

One species, the shiny cowbird, was excluded because home range size is arguably a poor proxy for cognitive demand due to its unique ecology. During their breeding season, female cowbirds locate nests to parasitize in the future so that host species take care of their eggs [106,107]. This requires remembering many different locations. Males do not do this, and it has been argued this explains why female cowbirds have better performance on spatial tasks than males. However, males have larger home ranges [107]. Thus, the shiny cowbird serves as a strong data point against the sex-specific adaptation hypothesis, as framed here using home range size as the proxy for cognitive demand. Although it is debatable whether remembering the locations of the nests they intend to parasitize is more cognitively demanding than navigating a larger home range, we opted to be conservative in our test of the sex-specific adaptation hypothesis and therefore excluded this species from our analyses.

Methods for measuring spatial ability are more diverse. Most non-human cognitive data came from maze tasks, primarily the Morris water maze, radial arm maze, T-maze or variants. Additional measurements included an ad hoc location memory task for the chimpanzee, homing ability after translocation in three frog species, an ad hoc measure of visuospatial ability in horses, and the delayed recognition span test for the rhesus monkey.

In humans, we limited data to measurements that included virtual or real-world wayfinding rather than proxies such as the mental rotation task. As noted earlier, artificial proxies show larger sex differences than tasks requiring actual navigation, which would inflate the human cognitive sex difference index.

Cognitive data from subsistence cultures consisted of pointing test measurements, in which participants must point to an out-of-sight location and angular deviation is measured. Data from non-subsistence cultures primarily came from the Nazareth *et al*. [19] meta-analysis. We used the following exclusions to filter studies (each exclusion refers to one of the categories the studies were classified by): task goal of ‘Reading maps (e.g. navigate by reading a map: Maps)’, task goal of ‘Navigating with verbal instructions (e.g. navigate with verbal directions: Verbal instructions)’, ‘Survey [perspective] (e.g. flying over a city, reading a map)’ and ‘Both [route and survey perspective] (e.g. using a map to walk through a city)’. The relationship between map reading and real-world navigation is uncertain [1], and a survey perspective is not representative of actual navigational tasks. We also decided that navigating with verbal instructions was an inferior measure of spatial ability when considering the many studies in which participants had to navigate without instructions. The latter is more representative of the demands of evolutionarily relevant situations in which wayfinding ability would be advantageous. After these exclusions, we selected both virtual and real-world tasks that involved the deliberate traversal of space, which we considered most representative of navigation demands in an ecological setting, and had the largest sample sizes. Studies in which the participants experienced a first-person view of another person navigating (e.g. from a video) were excluded. For a complete list of home range and spatial ability measurements included in the analysis, see electronic supplementary material, table S1.

Several measurements came directly from the Clint *et al*. [1] study. Despite criticism from Cashdan & Gaulin [10], we retained data from the rhesus monkey, horse, Talas tuco-tuco and cuttlefish. See electronic supplementary material, file S1 for the rationale of each inclusion.

### 2.2. Sex difference index

As in Clint *et al*. [1], we calculated a unitless index to reflect the sex difference for each measurement to facilitate averaging and comparisons among studies. The index was calculated in the same way as in the 2012 analysis. The quantity [(*X_g_*/*X_l_*)−1] was used, where *X_g_* is the mean of the sex with the greater value, and *X_l_* is the mean of the sex with the lesser value, a standard formula established in the literature for comparative analyses (e.g. [108–111]). If females display an advantage in spatial ability or a larger home range, then the quantity is multiplied by −1 to assign a negative value. Subtracting one from the ratio centres the measurements at zero and equalizes the range of possibilities for male and female values, such that a ratio of 1 : 1 would result in a sex difference index of zero. Each species was given one sex difference index for home range and one for spatial ability, equal to the average of the indices of each measurement for that species in each respective category (see electronic supplementary material, table S1).

### 2.3. Statistical analysis

All statistical analysis was performed in R (version 4.2.2). We first examined the correlation between sex differences in home range size and spatial ability on the species data points using phylogenetic generalized least-squares regression, a well-established statistical method that takes into account potential phylogenetic non-independence of species data points (e.g. [112–115]). This was accomplished using the base ‘gls’ function and specifying the variance–covariance structure as ‘corMartins’, ‘corGrafen,’ ‘corPagel’ or ‘corBrownian’ from the package ‘ape’. In addition, phylogenetic signal (‘K statistic’ following Blomberg *et al*. [116]) was estimated using the ‘phylosig’ function in the ‘phylotools’ package. The likelihoods of the models correcting for phylogeny were lower than ordinary least-squares (OLS) regressions, and phylogenetic signal was not significant (see Results). Thus, we report only results from ordinary and weighted least squares linear regression analyses.

Because species varied in the amount of data available, in addition to ordinary OLS, we also performed a weighted regression for each analysis using the total sample size as the weights. The sample size for each species was the sum of the number of unique subjects in each home range and spatial ability measure. If a subject gave data for multiple measurements, they were only counted once. Data from commentaries or methods that did not measure individual subjects did not contribute to the species sample size. This included some data for horses [62,63], humans [23,74], Natal mole-rats [88] and rhesus monkeys [99]. The sample sizes for one non-subsistence human range study and one spatial ability study are slightly inflated because it is unclear how many of the participants contributed to each specific measurement [65,67]. Home range data from one meadow vole study [81] and one prairie vole study [89] did not contribute to the sample size because the authors did not report the number of unique subjects. All of the values used in the analyses are available in electronic supplementary material, table S1.

For the main analyses, we used data from subsistence cultures to represent the human data point, reasoning that data from non-subsistence cultures poorly represent conditions of our evolutionary ancestry and are heavily biased toward a ‘Westernized’ culture. We also performed analyses with statistical outliers removed (see Results) and using the logarithm base 10 of the sex difference indices to account for positive skewness associated with ratios. For these analyses, the logarithm of the index + 1 was used to account for cases when the index was zero. For negative index values, the logarithm was taken of the absolute value of the index + 1 and then multiplied by negative 1.

While the main analyses used subsistence data for humans for reasons described above, we also analysed the data using non-subsistence measures for humans for reference, and included analyses that combined both subsistence and non-subsistence data (electronic supplementary material, table S2). We also included analyses with only non-human animals. Finally, for the main analyses described above, when raw data or means were unavailable for a particular study, but the sex difference was not significant, the sex difference index was set to zero. However, we also included analyses where those studies that did not report effect sizes were excluded from the calculation of the indices (electronic supplementary material, table S2, zeros removed). This resulted in the loss of two species (otter and tuco-tuco). All the data are available in electronic supplementary material, table S1 for further analyses.

To help interpret results of OLS models, we performed a statistical power analysis. We simulated data varying the correlation from 0 to 1 and then evaluated the proportion of simulations where the correlation was significant (i.e. *p* < 0.05, two-tailed test), using both weighted and unweighted OLS. Specifically, we used the ‘rbvnorm’ function from the ‘extraDistr’ package to generate 1000 simulated values from a bivariate normal distribution after specifying a correlation, and the mean and standard deviation of the *x* and *y* variables from the mean and standard deviation of the observed values. This was repeated for a sequence of correlations ranging from 0 to 1 in 0.01 increments (see electronic supplementary material, file S2 for the R-code used).

## 3. Results

**Figure 1.**
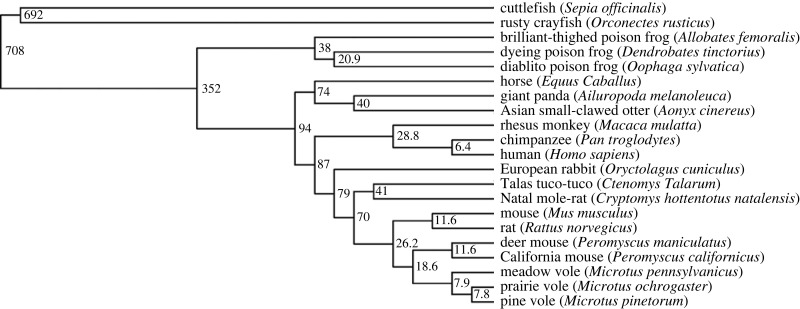
Phylogeny of the species used in the comparative analysis. The numbers at the bifurcations are estimated divergence times in millions of years. The branch lengths are not drawn to scale. The figure was generated using the Interactive Tree of Life online tool [117].

An initial test of phylogenetic signal in the home range and spatial navigation sex difference indices using the K statistic of Blomberg *et al*. [116] indicated small values (0.08 and 0.03, respectively) that were not significant (*p* = 0.23 and *p* = 0.89, respectively) based on randomization tests when using the phylogenetic tree with divergence times as branch lengths (figure 1). In addition, the diagnostic plots in Mesquite PDAP (from [118,119]) indicated that the phylogeny and/or branch lengths did not fit the tip data well. Finally, a 'phylomorphospace' plot [120–122] did not indicate any obvious relation of the phenotypes to phylogenetic position.

In the phylogenetically corrected regression analyses, the likelihood of models with phylogenetically informed variance–covariance matrices were substantially lower than those of the OLS models that do not correct for phylogenetic signal. Adding estimation of Grafen's *ρ* transformation of branch lengths [118] returned a very low estimate, indicating essentially a star phylogeny, and the same was true for Pagel's *λ*. Attempts to apply the accelerated/decelerated (ACDC) model of Blomberg *et al*. [116] or the transformation of Martins & Hansen [123] did not converge. Therefore, we believe the OLS estimates are appropriate for these analyses. Note, though, that we are limited by the taxa for which data are available. If future research that includes additional species detects significant phylogenetic signal, phylogenetic correction may be warranted.

**Figure 2.**
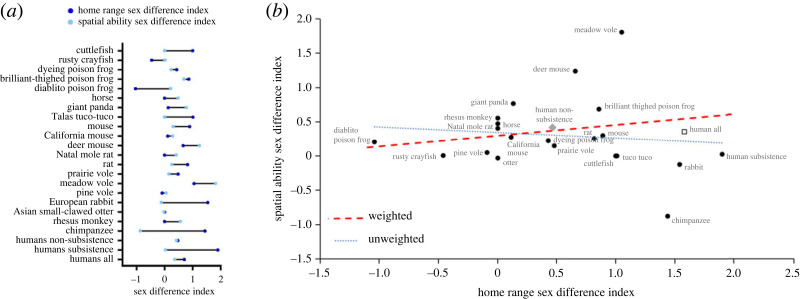
Sex differences in home range and spatial navigation across species. (*a*) Forest plot. (*b*) Scatterplot. The trendlines were generated using the subsistence data only for humans. The dashed red trendline shows the slope from the weighted least squares regression and the dotted blue trendline shows the slope for the unweighted regression. The values for non-subsistence and combined non-subsistence and subsistence are also shown as separate symbols but were not included in the trendline calculations.

**Figure 3.**
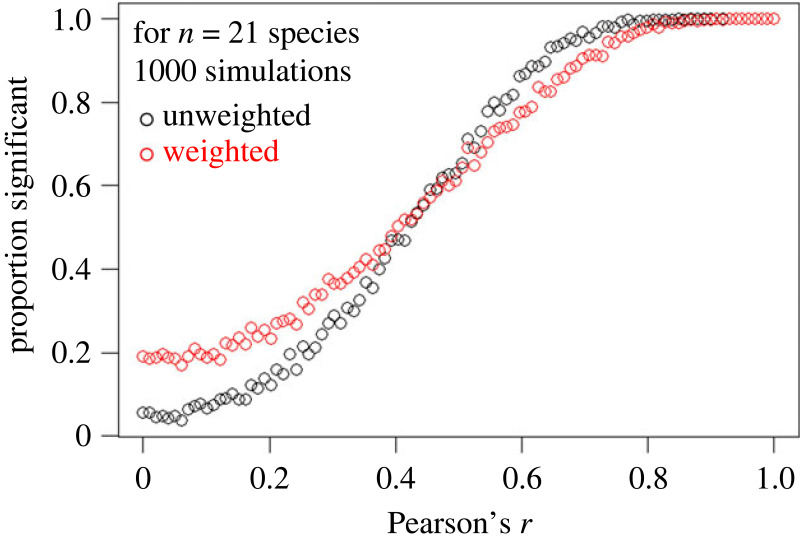
Statistical power analysis. Data were simulated from a bivariate normal distribution using the means and standard deviations of the home range and spatial ability sex difference indices from the 21 species, using the subsistence value for humans, and a sequence of correlations ranging from 0 to 1 in 0.01 increments. One thousand datasets were simulated for each correlation increment. For each dataset, we fit both a weighted and unweighted least-squares regression model and evaluated whether the correlation was significant (*p* < 0.05). The proportion of significant correlations out of the 1000 datasets is plotted on the *y*-axis against the sequence of correlation values used to generate the simulated datasets.

In the OLS analyses, the correlation between sex differences in home range size and spatial navigation was not statistically significant in any of our analyses that used subsistence measures for humans (table 1). The weighted analysis showed a weak positive correlation (*r* = 0.21) between home range and spatial ability dimorphism, while the unweighted analyses showed a weak negative correlation (*r* = −0.11; table 1 and figure 2). This pattern was true whether or not outliers were removed, or using logarithms of the indices. The same pattern was observed for analyses that excluded zeros estimated from unreported, non-significant effect sizes (electronic supplementary material, table S2; zeros removed analyses). Results of the statistical power analysis suggest that our comparative analyses with 21 species is sufficiently powered to detect correlations greater than approximately 0.5 assuming *p* < 0.05 is significant and a two-tailed test (figure 3). Thus, if the ‘true’ correlation is within the estimated range in table 1, it will be difficult to detect without many more species.

**Table 1.** Statistical results from the linear regression models. All analyses in this table used subsistence data for humans. The table shows estimates of the correlation, slope and intercept, including standard errors, confidence intervals and *p*-values. Results of analyses that were weighted, unweighted, with and without outliers, and with and without logarithmic transformation of the sex difference indices are shown.

analysis	corr	s.e.	low CI	high CI	slope	s.e.	low CI	high CI	*p*-value	intercept	s.e.	low CI	high CI	*p*-value
all data, weighted	0.21	0.224	−0.23	0.64	0.16	0.172	−0.18	0.49	0.37	0.30	0.169	−0.03	0.63	0.092
all data, unweighted	−0.11	0.228	−0.55	0.34	−0.08	0.172	−0.42	0.25	0.64	0.35	0.148	0.06	0.64	0.029
chimp and meadow vole removed, weighted	0.08	0.242	−0.40	0.55	0.02	0.074	−0.12	0.17	0.76	0.18	0.072	0.04	0.32	0.022
chimp and meadow vole removed, unweighted	−0.13	0.240	−0.60	0.34	−0.06	0.117	−0.29	0.17	0.60	0.32	0.095	0.13	0.50	0.0037
all data, logarithm weighted	0.27	0.221	−0.16	0.70	0.18	0.150	−0.11	0.48	0.24	0.08	0.042	0.00	0.17	0.055
all data, logarithm unweighted	−0.12	0.228	−0.56	0.33	−0.09	0.173	−0.43	0.25	0.61	0.11	0.042	0.03	0.19	0.017
chimp and meadow vole removed, logarithm weighted	0.18	0.238	−0.28	0.65	0.06	0.081	−0.10	0.22	0.45	0.06	0.022	0.02	0.10	0.013
chimp and meadow vole removed, logarithm unweighted	−0.11	0.241	−0.58	0.36	−0.06	0.128	−0.31	0.19	0.66	0.11	0.029	0.05	0.16	0.0023

In contrast to the lack of significant slope, the intercept differed significantly from zero in most analyses, and all estimates were positive (0.06–0.35), suggesting that, among species, males slightly outperform females, though not in any pattern related to home range. Only one species showed a significant female advantage, while the other 20 either showed no difference or a significant male advantage. The average sex difference in spatial navigation across all 21 species (using the subsistence value for humans) was 0.31 (± 0.117 s.e.; Cohen's *d* = 0.57). Figure 2 shows all the species data points and two linear relations, one weighted by the sample size for the species measurement, the other unweighted.

Two statistical outliers were identified as showing standardized residuals beyond 2 s.d. from the mean: the meadow vole and the chimpanzee. The chimpanzee was the only species with a significant female advantage in spatial ability, and the data came from a small sample (including only seven females and five males) from a single paper using a proxy for wayfinding. The meadow vole sex difference in spatial ability was the largest of all the species and came from an average of two studies using standard measurements and reasonable sample sizes (including 19 females and 15 males across two studies).

Electronic supplementary material analyses in which non-subsistence human data were included showed more moderate positive correlations in most cases significant when weighted, particularly when subsistence human data were excluded, and weak negative correlations when unweighted. Logarithmic transformation of the data did not change the overall pattern. When humans were removed altogether and data were weighted, a slightly significant positive correlation was detected (*p* = 0.047), but this went away when the outliers were removed. For unweighted non-human animal data, the slope was weakly negative (electronic supplementary material, table S2).

## 4. Discussion

We find little evidence that sex differences in home range size are correlated with sex differences in wayfinding across the available data for 21 species. When using the subsistence measures for humans, none of the analyses showed a significant correlation. Only when analyses were weighted by sample size and we included the huge sample size of non-subsistence measures (i.e. mostly industrial) was the among-species correlation statistically significant. When human data were removed from the analysis, we also observed a small, significant correlation, but this was driven by statistical outliers, because when they were removed, the correlation went away. Thus, the sex-specific adaptation hypothesis is not strongly supported, and non-adaptation alternatives should be seriously considered. The lack of significant correlation in most of our analyses (table 1; electronic supplementary material, table S2) builds on that of Clint *et al*. [1] and is what would be expected given basic evolutionary considerations. To date, no evidence of sexually antagonistic selection has been presented, only some gesturing toward the energy costs of maintaining navigation abilities for females [13,14].

Sex differences in behaviour or performance can arise from biological or cultural processes that have little to do with evolution. The brain is renowned for its plasticity. Experience-induced restructuring is the *sine qua non* of brain function at many scales, from the sub-synaptic to whole neural circuits [124,125]. In the literature on sex differences, insufficient attention is accorded to this basic feature of the brain, a central theme of modern neurobiology, as well as phenotypic plasticity in general, a central theme of evolutionary biology. Phenotypic plasticity is the obvious null hypothesis to use in evaluating claims that any putative male superiority in spatial navigation is a sex-specific cognitive adaptation.

For humans, sex-specific experience has an obvious socio-cultural dimension. Evolutionary psychologists consider a behavioural trait innate if it is culturally universal. This is not the place to comment on that practice generally, but it is not sufficient to compare American and Chinese undergraduate psychology students, as in Geary & DeSoto [3], because they are too similar in the way they are socialized. Recent evidence in subsistence populations strongly suggests that sex difference in spatial navigation in humans is not a cultural universal. Rather, it disappears in cultures where males and females have similar ranging behaviour [24,25,36,37,39]. We believe that future research on human sex differences in navigation should focus on the role of socialization and culture, rather than evolutionary genetic factors.

In animals where males and females are constrained to have the same experience within their lifespan, ruling out the possibility for differential phenotypic plasticity, sex differences in wayfinding could result simply as a side effect of sex-specific aspects of reproductive physiology or development, i.e. the spandrel hypothesis. Some evidence suggests that androgens enhance spatial navigation in humans and other animals (e.g. [126–129]), but no consensus exists, especially with respect to activational effects (see [130,131]). Further, a handful of studies suggest that high levels of oestradiol decrease spatial navigation performance in rodents and humans, particularly when comparing the performance of females during the peaks and valleys of oestradiol concentrations, such as during the oestrus or menstrual cycle [79,132–134]. The significant intercepts observed in the majority of analyses (table 1; electronic supplementary material, table S2) suggest males generally outperformed females across species independent of sex differences in home range size. This result is consistent with but does not require androgenic or oestrogenic effects on spatial cognition. Whether sex-hormone related or not, we find little evidence that the small male advantage in wayfinding results from natural (or sexual) selection. Hence, our data do not favour the sex-specific adaptation hypothesis over the spandrel hypothesis, according to which these sex differences are unselected by-products that are unrelated to the putative ecological, evolutionary driver—the sex difference in home range. Although the data perhaps could be explained by alternative adaptation hypotheses unrelated to home range, non-adaptive explanations such as the spandrel hypothesis deserve at least equal consideration. In our opinion, it is important not to assume *a priori* that adaptation hypotheses are more likely than other explanations, such as side effects of sex physiology.

Over the past half-century, significant resources have gone into testing the sex-specific adaptation hypothesis as an explanation for sex differences in navigation abilities. In a previous meta-analysis, we found the evidence was weak, and in this paper with an expanded dataset, we again find little evidence supporting the sex-specific adaptation hypothesis. The data that we had to work with are limited in terms of the small number of species for which both home range sex differences and spatial navigation were measured. Further, there is the potential for a large amount of noise in the species data points for the sex difference index because of individual variability and measurement error in the numerator and denominator of the ratios. It is possible with more, higher quality data, a significant positive relationship will appear. To date, the observations, such as they are, do not favour the sex-specific adaptation hypothesis over the alternatives we have described. We conclude that non-adaptive explanations for sex differences in navigation in humans and other animals should be taken more seriously. More broadly, we strongly believe the fields of evolutionary psychology and behavioural ecology would benefit from increased consideration of non-adaptive explanations in their endeavours to explain the origin of variation in phenotypic traits.

## Data Availability

All data come from previously published manuscripts. We include an electronic supplementary material table with all the data values with source references [135].
